# The impact of prescription opioids on all-cause mortality in Canada

**DOI:** 10.1186/s13011-016-0071-4

**Published:** 2016-08-01

**Authors:** Sameer Imtiaz, Jürgen Rehm

**Affiliations:** 1 Institute of Medical Science, University of Toronto, Toronto, Canada; 2 Social and Epidemiological Research Department, Centre for Addiction and Mental Health, Toronto, Canada; 3 Campbell Family Mental Health Research Institute, Centre for Addiction and Mental Health, Toronto, Canada; 4 Department of Psychiatry, University of Toronto, Toronto, Canada; 5 Institute for Clinical Psychology and Psychotherapy, Technische Universität Dresden, Dresden, Germany; 6 Dalla Lana School of Public Health, University of Toronto, Toronto, Canada

**Keywords:** Canada, All-cause mortality, Drug poisoning mortality, Opioid analgesics

## Abstract

An influential study from the United States generated considerable discussion and debate. This study documented rising morbidity and mortality in midlife among white non-Hispanic Americans in the 21st century, with clear linkages of all-cause mortality to increasing rates of poisonings, suicides and chronic liver disease deaths. All of these causes of deaths are strongly related to the use of legal and illegal substances, but the study stressed the importance of prescription opioids. Given the similarities between the United States and Canada in prescription opioid use, the assessment of similar all-cause mortality trends is relevant for Canada. As this commentary highlights, the all-cause mortality shifts seen in the United States cannot be seen in Canada for either sex or age groups. The exact reasons for the differences between the two countries are not clear, but it is important for public health to further explore this question.

**Electronic supplementary material:**

The online version of this article (doi:10.1186/s13011-016-0071-4) contains supplementary material, which is available to authorized users.

## Background

One of the most influential studies in public health in the United States (US) in 2015 highlighted a reversal of all-cause mortality decreases in midlife among white non-Hispanic Americans in the 21st century [1]. This study triggered numerous newspaper reports, as well as debate [2–4]. It showed a clear link of all-cause mortality in this group to increasing rates of poisoning, suicide and chronic liver disease deaths, all of which are strongly related to use of legal and illegal substances [5]. The non-medical use of prescription opioids (POs) was especially stressed in the study as the underlying risk factor for the all-cause mortality increases [1]. Poisoning deaths related to POs, often referred to as overdose deaths, have been strongly linked to the overall level of PO use, with a near perfect correlation of 0.99 [6].

## Main text

The study described above by Case and Deaton (2015) raises the question about similar all-cause mortality trends in Canada. Canada is second to the US in overall use of POs [7], but while the US has decreased their level of *per capita* use for the first time, Canada again increased it. Moreover, non-medical use of POs is relatively similar between the two countries. For example, non-medical use of POs reached 7.7 % in 2010 for Ontario [8], whereas the analogous estimate in the US was 5.5 % [9]. It should be noted, however, that non-medical use of POs’ prevalence vary in both countries, with variability strongly associated with exact phrasing of relevant questions. Regarding other substance use, alcohol consumption *per capita* is higher in Canada than the US (10.2 L ethanol vs. 9.2 L ethanol) [10], whereas past 12-month cocaine use is lower (1.3 % vs. 2.2 %) [11], as is lifetime heroin use (0.5 % vs. 1.8 %) [12, 13]. Although the corresponding 12-month heroin use estimate is not available for Canada, there is no reason to believe that it would be higher than that observed for the US.

Case and Deaton (2015) included Canada in their comparisons with the US midlife age group (45–54 years), and found no such increase in all-cause mortality for the midlife age group in Canada, but rather a decrease. The present commentary extended similar assessments to other age groups as well. Based on the World Health Organization Mortality Database and World Population Prospects Database [14, 15], all-cause mortality rates were calculated by sex and 10-year age-groups. Changes in all-cause mortality rates were thereafter calculated for each successive decade from 1990 until 2011. Apart from changes in all-cause mortality rates, Pearson’s product moment correlations were computed between all-cause mortality rates and drug poisoning mortality rates (ICD-10 X40-X44, X60-X64, X85 and Y10-Y14) by sex from 2000 to 2011. All mortality rates were standardized according to the Canadian population in 1990.

The all-cause mortality rates for males decreased from 758 deaths per 100,000 in 1990 to 654 deaths per 100,000 in 2000 to 528 deaths per 100,000 in 2011, indicating decreases of 104 deaths per 100,000 between 1990 and 2000 and 126 deaths per 100,000 between 2000 and 2011. The all-cause mortality rates for females also decreased in the examined time period, with decreases of 5 deaths per 100,000 (from 631 deaths per 100,000 to 626 deaths per 100,000) and 64 deaths per 100,000 (from 626 deaths per 100,000 to 562 deaths per 100,000) between 1990 and 2000 and 2000 and 2011, respectively. The all-cause mortality rates by age groups are presented in Table 1 for males and Table 2 for females. As evident by these tables, all-cause mortality rates decreased between 1990, 2000 and 2011 for both sexes and all age groups, with the exception of females 65 years and older between 1990 and 2000.

**Table 1:** All-cause mortality rates for males from 1990–2011 in Canada

Year	Deaths per 100,000						
	<15 Years	15–24 Years	25–34 Years	35–44 Years	45–54 Years	55–64 Years	≥65 Years
1990	81	103	124	190	447	1277	5412
2000	50	78	90	157	354	962	4937
Δ1990–2000	31	25	34	33	93	315	475
2000	50	78	90	157	354	962	4937
2011	48	61	74	123	296	752	3987
Δ2000–2011	2	17	17	34	58	211	950

**Table 2:** All-cause mortality rates for females from 1990–2011 in Canada

Year	Deaths per 100,000						
	<15 Years	15–24 Years	25–34 Years	35–44 Years	45–54 Years	55–64 Years	≥65 Years
1990	63	35	50	100	262	674	3880
2000	39	34	41	91	229	573	3994
Δ1990–2000	24	1	9	9	34	101	−115
2000	39	34	41	91	229	573	3994
2011	39	27	38	75	205	468	3623
Δ2000–2011	0	7	3	17	23	105	371

The real decreases in all-cause mortality rates should be higher, given that the average age within the age groups has been increasing (for the effect in the US see [4]) [16]. This latter effect is likely also responsible for the one exception in trends among the oldest females during the first decade of observation.

**Fig. 1:**
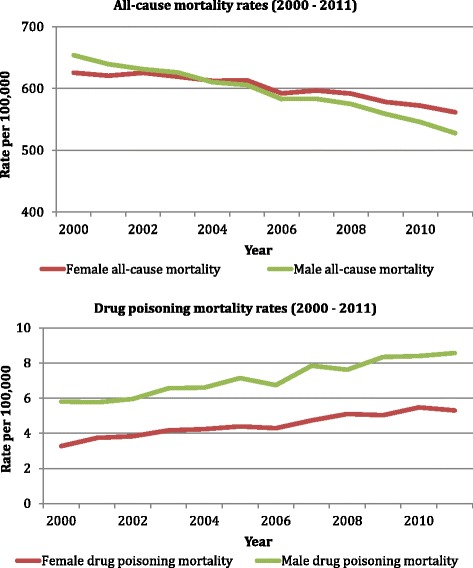
All-cause mortality rates (top panel) and drug poisoning mortality rates (bottom panel) by sex (2000–2011)

The sex-specific all-cause mortality rates and drug poisoning mortality rates from 2000 to 2011 are displayed in Fig. 1 (see Additional file 1: Tables S1 and S2 for the data). Drug poisoning mortality rates increased for males and females during the examined time period, despite decreases in all-cause mortality rates for both sexes. As a consequence, strong negative correlations were observed between the two rates for males (r = −0.95, p < 0.01) and females (r = −0.91, p < 0.01). It follows that the increases in drug poisoning mortality rates were not large enough to drive up the all-cause mortality rates, as was observed by Case and Deaton for the US [1].

It should also be noted that although the drug poisoning mortality rates increased between 2000 and 2011 in Canada, these increases were considerably lower compared with those observed in the US. For example, drug poisoning mortality rates for those 45–54 years rose by 7 deaths per 100,000 and 6 deaths per 100,000 in Canada for males and females, respectively. The analogous increases in the US surmounted to 14 deaths per 100,000 for males and 16 deaths per 100,000 for females. Given the similarities in the use of legal and illegal substances between the two countries, the observed differences in these trends are noteworthy, but the exact reasons underlying these differences are not well understood.

## Conclusion

In sum, the upward all-cause mortality shifts seen in the US cannot be seen in Canada for either sex or age groups, even though Canada has also been experiencing increases in PO use, non-medical use of POs and related overdose deaths [17, 18]. The exact reasons for the differences between the two countries are not clear, but it is important for public health to further explore this question.

## Abbreviations

PO, Prescription opioid; US, United States

## Additional file

Additional file 1: Table S1. All-cause mortality rates for males and females in Canada (2000–2011). **Table S2.** Drug poisoning mortality rates for males and females in Canada (2000–2011). (DOCX 43 kb)
