# Supplementary material for: The impact of prescription opioids on all-cause mortality in Canada
Source: Subst Abuse Treat Prev Policy. 2016 Aug 1;11:27. doi: 10.1186/s13011-016-0071-4 (PMC4968006; doi:10.1186/s13011-016-0071-4)
Supplement: Additional file 1: Table S1. — All-cause mortality rates for males and females in Canada (2000–2011). Table S2. Drug poisoning mortality rates for males and females in Canada (2000–2011). (DOCX 43 kb) [file 13011_2016_71_MOESM1_ESM.docx]

**Table S1: All-cause mortality rates for males and females in Canada (2000 – 2011)**

|  |  |  |
| --- | --- | --- |
| **Year** | **Deaths per 100,000** | |
|  | **Male** | **Female** |
| 2000 | 654 | 626 |
| 2001 | 639 | 621 |
| 2002 | 631 | 625 |
| 2003 | 626 | 619 |
| 2004 | 610 | 612 |
| 2005 | 606 | 613 |
| 2006 | 583 | 592 |
| 2007 | 583 | 597 |
| 2008 | 575 | 592 |
| 2009 | 559 | 578 |
| 2010 | 546 | 572 |
| 2011 | 528 | 562 |

**Table S2: Drug poisoning mortality rates for males and females in Canada (2000 – 2011)**

|  |  |  |
| --- | --- | --- |
| **Year** | **Deaths per 100,000** | |
|  | **Male** | **Female** |
| 2000 | 6 | 3 |
| 2001 | 6 | 4 |
| 2002 | 6 | 4 |
| 2003 | 7 | 4 |
| 2004 | 7 | 4 |
| 2005 | 7 | 4 |
| 2006 | 7 | 4 |
| 2007 | 8 | 5 |
| 2008 | 8 | 5 |
| 2009 | 8 | 5 |
| 2010 | 8 | 5 |
| 2011 | 9 | 5 |
